# Aging Additively Influences Insulin- and Insulin-Like Growth Factor-1-Mediated Endothelial Dysfunction and Antioxidant Deficiency in Spontaneously Hypertensive Rats

**DOI:** 10.3390/biomedicines9060676

**Published:** 2021-06-15

**Authors:** Kunanya Masodsai, Yi-Yuan Lin, Sih-Yin Lin, Chia-Ting Su, Shin-Da Lee, Ai-Lun Yang

**Affiliations:** 1 Faculty of Sports Science, Chulalongkorn University, Bangkok 10330, Thailand; kunanya.m@chula.ac.th; 2 Institute of Sports Sciences, University of Taipei, Taipei 11153, Taiwan; szu1015@gmail.com; 3 Department of Exercise and Health Science, National Taipei University of Nursing and Health Sciences, Taipei 11257, Taiwan; yiyuanlin@ntunhs.edu.tw; 4 Department of Occupational Therapy, College of Medicine, Fu Jen Catholic University, New Taipei City 24205, Taiwan; chiatingsu@gmail.com; 5 Department of Physical Therapy, Asia University, Taichung 41354, Taiwan; leeshinda@gmail.com; 6 Department of Physical Therapy, Graduate Institute of Rehabilitation Science, China Medical University, Taichung 40402, Taiwan; 7 School of Rehabilitation Medicine, Weifang Medical University, Shandong, Weifang 261000, China

**Keywords:** aging, hypertension, endothelium, antioxidant activity, oxidative stress

## Abstract

This study aimed to investigate the aging-related endothelial dysfunction mediated by insulin and insulin-like growth factor-1 (IGF-1) and antioxidant deficiency in hypertension. Male spontaneously hypertensive rats (SHRs) and age-matched normotensive Wistar–Kyoto rats (WKYs) were randomly divided into 24-week-old (younger) and 48-week-old (older) groups, respectively. The endothelial function was evaluated by the insulin- and IGF-1-mediated vasorelaxation of aortic rings via the organ bath system. Serum levels of nitric oxide (NO), malondialdehyde (MDA), catalase, and total antioxidant capacity (TAC) were examined. The insulin- and IGF-1-mediated vasorelaxation was significantly impaired in both 24- and 48-week-old SHRs compared with age-matched WKYs and was significantly worse in the 48-week-old SHR than the 24-week-old SHR. After pretreatments of phosphoinositide 3-kinase (PI3K) or NO synthase (NOS) inhibitors, the insulin- and IGF-1-mediated vasorelaxation became similar among four groups. The serum level of MDA was significantly increased, while the NO, catalase, and TAC were significantly reduced in the 48-week-old SHR compared with the 24-week-old SHR. This study demonstrated that the process of aging additively affected insulin- and IGF-1-mediated endothelial dysfunction in SHRs, which could be partly attributed to the reduced NO production and antioxidant deficiency.

## 1. Introduction

Hypertension-related morbidity and mortality have been increasing in the elderly [1]. Aging has remarkable influences on the decline of cardiovascular function. It may subsequently contribute to the occurrence of cardiovascular diseases (CVD), such as hypertension, atherosclerosis, and myocardial infarction [2,3]. Epidemiological studies have indicated that advancing age is associated with the prevalence of hypertension, and more than half of the elderly population have hypertension. Specifically, hypertension leads to pathological changes of vascular function, including vascular hypertrophy and remodeling, reduced lumen diameter, vascular smooth muscle cell (VSMC) proliferation, as well as endothelial dysfunction [4,5,6]. Endothelial dysfunction is mainly characterized by the impaired nitric oxide (NO) bioavailability, diminished endothelium-induced vasorelaxation, and excessive generation of oxidative stress [7,8]. Reactive oxygen species (ROS) and oxidative changes of signaling molecules have been reported as strongly associated with the development of CVD, such as hypertension and atherosclerosis. The process of aging is also related to the increased production of oxidative stress, which may result in the development of endothelial dysfunction and CVD. Moreover, antioxidant deficiency could be one of the key mechanisms mediating the reduced NO bioavailability and impaired endothelium-dependent vasorelaxation in aging and hypertension, respectively [9,10,11,12,13].

Previous studies have reported that insulin and insulin-like growth factor-1 (IGF-1) play important roles in the modulation of endothelial function, which stimulate NO production and consequently evoke the endothelium-dependent vasorelaxation. They both induce NO production by activating phosphatidylinositol 3-kinase (PI3K), protein kinase B (PKB/Akt), and endothelial NO synthase (eNOS) [14,15]. The vascular actions of insulin and IGF-1 have been found impaired in some cardiovascular disorders, such as hypertension and obesity. In addition, the increased level of ROS production may contribute to the impairments of insulin- and IGF-1-mediated vasorelaxation [16,17]. Our previous study indicated that the administration of superoxide dismutase (SOD) attenuated the insulin- and IGF-1-mediated vascular dysfunction in spontaneously hypertensive rats (SHRs). This implied that the reduced level of antioxidants might involve in the impairments of insulin- and IGF-1-mediated vasorelaxation in hypertension [18]. However, whether the process of aging additively influences insulin- and IGF-1-mediated endothelial dysfunction and antioxidant deficiency in hypertension and its underlying mechanisms remained unclear. Therefore, this study aimed to investigate the aging-related insulin- and IGF-1-mediated endothelial dysfunction and antioxidant deficiency in SHRs.

## 2. Materials and Methods

### 2.1. Experimental Animals

The spontaneously hypertensive rats (SHRs) are well recognized as a model of hypertension with an increase in peripheral vascular resistance, developing high blood pressure and impaired cardiac and vascular functions [19]. The age-matched Wistar–Kyoto rats (WKYs) were provided as the normotensive control groups. Male SHRs and WKYs were purchased from the National Laboratory Animal Center (Taipei, Taiwan). The SHRs were randomly divided into two age groups, the 24-week-old (24wk; younger) and 48-week-old (48wk; older) groups (24wk-SHR and 48wk-SHR), while the WKY rats were served as the age-matched normotensive groups (24wk-WKY and 48wk-WKY). All experimental procedures were approved by the Institutional Animal Care and Use Committee of the University of Taipei, Taiwan (Ethical approval code: UT105005, 6 February 2017), and conducted in conformity under the Guide for the Care and Use of Laboratory Animals of the National Institutes of Health.

At the age of 24 and 48 weeks old, the heart rate (HR), systolic/diastolic blood pressure (SBP/DBP), and mean arterial pressure (MAP) were monitored in conscious rats by the tail-cuff method (BP98A, Softron Co., Ltd., Tokyo, Japan).

### 2.2. Determination of Blood Glucose and Insulin Resistance

The glucose oxidase method was used to determine fasting blood glucose by using the glucometer (Roche Diagnostics, Indianapolis, IN, USA). Serum insulin was analyzed using a commercial enzyme-linked immunosorbent assay (ELISA) kit (Mercodia AB, Uppsala, Sweden) according to the manufacturer’s instructions. Briefly, the enzyme conjugate solution was incubated with the serum sample, and then the bound conjugate was detected by the reaction with 3,3′-5,5′-tetramethylbenzidine (TMB). The reaction was stopped by the stop solution, and the absorbance at 450 nm was measured by a microplate reader (TECAN Infinite M200PRO, Grödig, Austria). The homeostatic model assessment of insulin resistance (HOMA-IR) was computed as follows: (fasting glucose (mmol/L) × insulin (mU/L)/22.5) [20].

### 2.3. Measurement of Vasorelaxation

At the age of 24 and 48 weeks old, the rats were sacrificed under anesthesia with 2% isoflurane delivered in oxygen (95% O_2_ and 5% CO_2_), and then thoracic aortas were carefully isolated. The aortic rings were submerged in organ chambers containing the Krebs–Ringer buffer (118 mmol/L NaCl, 4.8 mmol/L KCl, 2.5 mmol/L CaCl_2_, 1.2 mmol/L MgSO_4_, 1.2 mmol/L KH_2_PO_4_, 24 mmol/L NaHCO_3_, 0.03 mmol/L Na-EDTA, and 11 mmol/L glucose; pH 7.4) oxygenated with 95% O_2_ and 5% CO_2_ at 37 °C. Then, aortic rings were monitored isometrically by force displacement transducers (Models FT3E, Grass Instrument, West Warwick, RI, USA) to evaluate the vasorelaxant responses. Before the drug administration, the equilibration at the optimal passive tension (i.e., 2 g) of aortic rings were completed for 60 min. After equilibration, aortic rings were pre-contracted by phenylephrine (10^−7^ mol/L, Sigma Chemical, St. Louis, MO, USA) and exposed to various concentrations of insulin (3 × 10^−8^ to 3 × 10^−6^ mol/L, Sigma Chemical, St. Louis, MO, USA) and IGF-1 (10^−9^ to 10^−7^ mol/L, PeproTech, Rocky Hill, NJ, USA) to induce dose-dependent vasorelaxant responses. In some of the aortic rings, selective inhibitors of vasorelaxation, nitro-L-arginine methyl ester (L-NAME) (10^−6^ mol/L; a potent NOS inhibitor; Sigma Chemical, St. Louis, MO, USA) and wortmannin (3 × 10^−7^ mol/L; a PI3K inhibitor; Sigma Chemical, St. Louis, MO, USA), were pretreated for 15 min before the administration of phenylephrine. These inhibitors were used to investigate the roles of NOS and PI3K in the insulin- and IGF-1-mediated vasorelaxation.

### 2.4. Serum Levels of Nitrate/Nitrite (NO), Malondialdehyde (MDA), and Antioxidant Activities

The serum was carefully separated from blood samples after the centrifugation at 2000× *g* for 15 min at 4 °C and stored at −80 °C for biochemical analysis. Serum levels of nitrate/nitrite (NO), MDA, catalase activity, and total antioxidant capacity (TAC) were analyzed in duplicate following the standard procedures with the manufacturer’s instructions by commercial ELISA kits. Briefly, serum NO concentration was analyzed by a nitrate/nitrite colorimetric assay kit (Cayman Chemical Company, Ann Arbor, MI, USA). Total nitrate/nitrite concentration was determined by two-step processes: (a) the conversion of nitrate to nitrite by nitrate reductase, and (b) the addition of Griess Reagents, which convert nitrite to a deep purple azo product. The absorbance at 450 nm due to the azo chromophore was measured by a microplate reader (TECAN Infinite M200PRO, Grödig, Austria). The serum MDA level, an index of lipid peroxidation marker, was determined by a thiobarbituric acid reactive substances (TBARS) assay kit (Cayman Chemical Company, Ann Arbor, MI, USA). The TBA reagent was mixed with the serum samples to generate the MDA-TBA adducts under high temperature (90–100 °C) and acidic conditions. After completing the reactions, samples were measured colorimetrically by a microplate reader (TECAN Infinite M200PRO, Grödig, Austria) at 540 nm. Serum catalase activity was measured by a catalase assay kit (Cayman Chemical Company, Ann Arbor, MI, USA). The assay was based on the reaction of catalase in the sample with methanol in the presence of hydrogen peroxide (H_2_O_2_). The 540-nm absorbance was read by a microplate reader (TECAN Infinite M200PRO, Grödig, Austria). Serum TAC was determined by an antioxidant assay kit (Cayman Chemical Company, Ann Arbor, MI, USA). The assay was based on the ability of antioxidants in the sample to inhibit the oxidation of the 2,2′-azino-di-(3-ethylbenzothiazoline sulphonate) (ABTS) to ABTS**^•⁺^** by metmyoglobin. The TAC in the sample was compared with that of Trolox (as the standard control) and quantified as millimolar (mmol/L) Trolox equivalents. The absorbance was measured colorimetrically at 750 nm by a microplate reader (TECAN Infinite M200PRO, Grödig, Austria).

### 2.5. Statistical Analysis

All data were presented as the means ± standard error of mean (SEM). One-way analysis of variance (ANOVA) was used to perform multiple group comparisons followed by LSD post hoc analysis using the SPSS software (Version 21, IBM, Armonk, NY, USA). On all occasions, *p* < 0.05 was considered statistically significant.

## 3. Results

### 3.1. General Characteristics

Table 1 shows that body weight was significantly increased with aging in the 48wk-WKY group and 48wk-SHR group compared with the 24wk-WKY group (*p* < 0.05) and 24wk-SHR group (*p* < 0.05), respectively. Both 24wk-SHR and 48wk-SHR groups showed significantly higher heart rate, blood pressure (i.e., SBP, DBP, and MAP), glucose, and HOMA-IR compared with the 24wk-WKY group (*p* < 0.05) and 48wk-WKY group (*p* < 0.05), respectively. Moreover, the 48wk-SHR group showed significantly higher insulin levels and HOMA-IR compared with the 24wk-SHR group (*p* < 0.05).

### 3.2. Insulin- and IGF-1-Mediated Vasorelaxation

**Figure 1:**
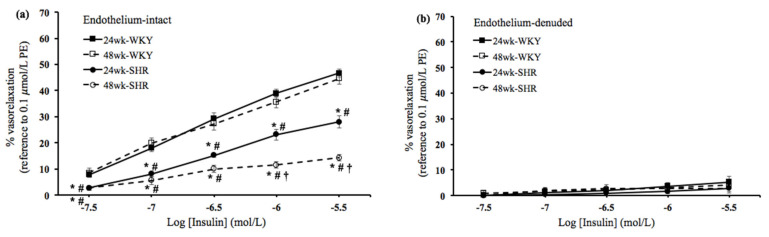
Insulin–mediated vasorelaxation. Insulin (3 × 10^−8^ to 3 × 10^−6^ mol/L)–mediated vasorelaxation at cumulative concentration–response curves in (**a**) endothelium–intact rings and (**b**) endothelium–denuded rings in the 24wk-WKY, 48wk-WKY, 24wk-SHR, and 48wk-SHR groups. The vasorelaxing responses were expressed as the percentages of the 0.1 μmol/L PE–induced precontractile force. Abbreviation: PE, phenylephrine. * *p* < 0.05, significant differences from 24wk-WKY; ^#^ *p* < 0.05, significant differences from 48wk-WKY; ^†^ *p* < 0.05, significant differences from 24wk-SHR; *n* = 8 in each group.

**Figure 2:**
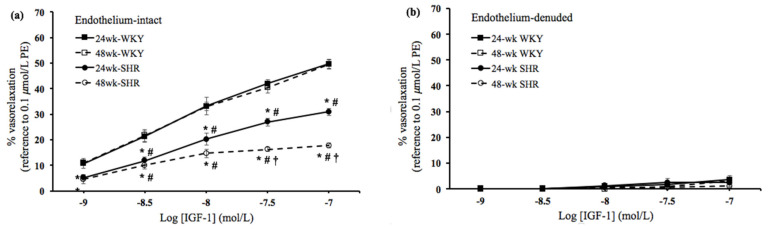
IGF-1–mediated vasorelaxation. IGF-1 (10^−9^ to 10^−7^ mol/L)–mediated vasorelaxation at cumulative concentration–response curves in (**a**) endothelium–intact rings and (**b**) endothelium–denuded rings in the 24wk-WKY, 48wk-WKY, 24wk-SHR, and 48wk-SHR groups. The vasorelaxing responses were expressed as the percentages of the 0.1 μmol/L PE–induced precontractile force. Abbreviation: PE, phenylephrine. * *p* < 0.05, significant differences from 24wk-WKY; ^#^ *p* < 0.05, significant differences from 48wk-WKY; ^†^ *p* < 0.05, significant differences from 24wk-SHR; *n* = 8 in each group.

In order to study the effects of the process of aging on vascular endothelial dysfunction in hypertension, the insulin- and IGF-1-mediated vasorelaxation was evaluated in endothelium-intact and denuded rings among the four groups. As shown in Figure 1a and Figure 2a, in the endothelium-intact rings, the insulin- and IGF-1-mediated vasorelaxation was significantly lower in either the 24wk-SHR group or the 48wk-SHR group than both of the WKY groups (*p* < 0.05). In addition, the 48wk-SHR group showed significantly worse insulin- and IGF-1-mediated vasorelaxation compared with the 24wk-SHR group (*p* < 0.05). However, no significant differences of vasorelaxation were found between the 24wk-WKY group and 48wk-WKY group. As shown in Figure 1b and Figure 2b, in the endothelium-denuded rings, the insulin- and IGF-1-mediated vasorelaxation was diminished, and no significant differences were found among the four groups.

### 3.3. Roles of NOS and PI3K in the Vasorelaxation Mediated by Insulin and IGF-1

**Figure 3:**
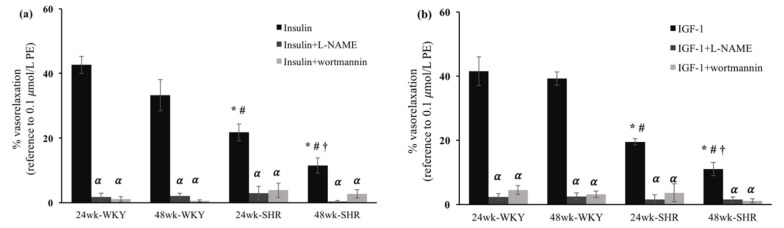
Vasorelaxation after the pretreatment of nitro-L-arginine methyl ester (L-NAME) and wortmannin. (**a**) Insulin (10^−6^ mol/L)-mediated vasorelaxation and (**b**) IGF-1 (3 × 10^−8^ mol/L)-mediated vasorelaxation after the pretreatment of L-NAME and wortmannin in the 24wk-WKY, 48wk-WKY, 24wk-SHR, and 48wk-SHR groups. The vasorelaxing responses were expressed as the percentages of the 0.1 μmol/L PE-induced precontractile force. Abbreviation: PE, phenylephrine.* *p* < 0.05, significant differences from 24wk-WKY; ^#^ *p* < 0.05, significant differences from 48wk-WKY; ^†^ *p* < 0.05, significant differences from 24wk-SHR; ^𝛼^ *p* < 0.05, significant differences from no inhibitors; *n =* 8 in each group.

To verify the roles of NOS and PI3K in the vasorelaxation mediated by insulin and IGF-1, selective inhibitors (i.e., L-NAME and wortmannin) were pretreated to blunt the vasorelaxation. Before L-NAME or wortmannin were added, either the 24wk-SHR group or the 48wk-SHR group showed significantly lower vasorelaxant responses to insulin (10^−6^ mol/L) and IGF-1 (3 × 10^−8^ mol/L) compared with both of the WKY groups (*p* < 0.05). Moreover, the 48wk-SHR group showed significantly worse vasorelaxation mediated by insulin and IGF-1 compared with the 24wk-SHR group (*p* < 0.05). After either L-NAME or wortmannin was added, the average levels of phenylephrine-induced pre-contraction responses were between 1.0 and 1.4 g and indicated no significant differences among the four groups. In addition, the vasorelaxant responses to insulin (10^−6^ mol/L) and IGF-1 (3 × 10^−8^ mol/L) were significantly diminished in the four groups, and no significant differences were found among the four groups (Figure 3a,b).

### 3.4. Serum Nitrate/Nitrite Concentration

**Figure 4:**
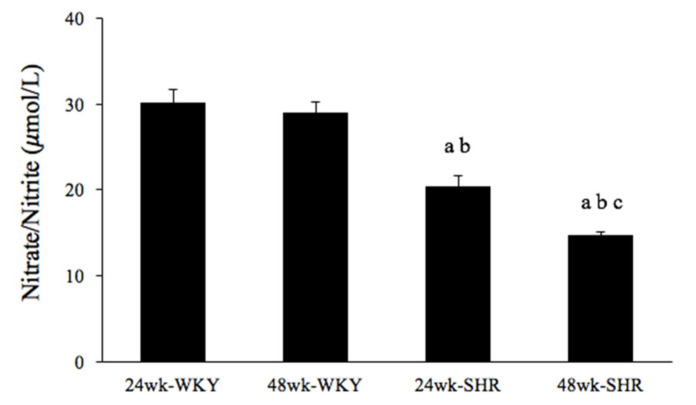
Serum nitrate/nitrite concentration in the 24wk-WKY, 48wk-WKY, 24wk-SHR, and 48wk-SHR groups. ^a^ *p* < 0.05, significant differences from 24wk-WKY; ^b^ *p* < 0.05, significant differences from 48wk-WKY; ^c^ *p* < 0.05, significant differences from 24wk-SHR; *n =* 8 in each group.

To detect effects of the process of aging on the NO production in hypertension, serum nitrate/nitrite concentration was evaluated among the four groups. Figure 4 shows that serum nitrate/nitrite concentration was significantly lower in either the 24wk-SHR group or the 48wk-SHR group than both of the WKY groups (*p* < 0.05). In addition, the 48wk-SHR group showed significantly reduced nitrate/nitrite concentration compared with the 24wk-SHR group (*p* < 0.05). However, there was no significant difference in the nitrate/nitrite concentration between the 24wk-WKY group and 48wk-WKY group.

### 3.5. Serum Levels of MDA, Catalase Activity, and TAC

**Figure 5:**
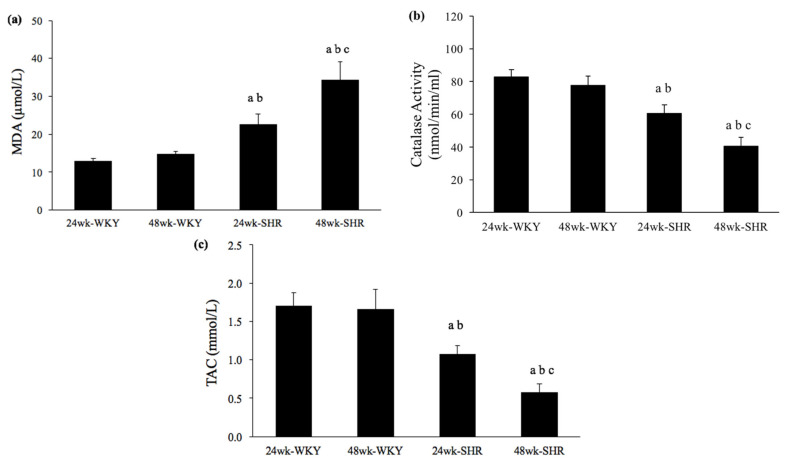
Serum levels of malondialdehyde (MDA), catalase activity, and total antioxidant capacity (TAC). (**a**) Serum MDA concentration, (**b**) catalase activity, and (**c**) TAC in the 24wk-WKY, 48wk-WKY, 24wk-SHR, and 48wk-SHR groups. ^a^ *p* < 0.05, significant differences from 24wk-WKY; ^b^ *p* < 0.05, significant differences from 48wk-WKY; ^c^ *p* < 0.05, significant differences from 24wk-SHR; *n* = 8 in each group.

To verify effects of the process of aging on oxidative stress and antioxidants in hypertension, serum levels of MDA, catalase activity, and TAC were evaluated among the four groups. As shown in Figure 5a, serum MDA concentration was significantly higher in either the 24wk-SHR group or the 48wk-SHR group than both of the WKY groups (*p* < 0.05). Furthermore, the 48wk-SHR group showed the highest MDA concentration among the four groups (*p* < 0.05). However, no significant differences of MDA concentration were found between the 24wk-WKY group and 48wk-WKY group. Figure 5b,c show serum levels of catalase activity and TAC, which represent antioxidant activities among the groups. Either the 24wk-SHR group or the 48wk-SHR group showed significantly lower catalase activity and TAC compared with both of the WKY groups (*p* < 0.05). Moreover, the 48wk-SHR group showed significantly impaired antioxidant activities compared with the 24wk-SHR group (*p* < 0.05). However, no significant differences of catalase activity and TAC were found between the 24wk-WKY group and 48wk-WKY group.

## 4. Discussion

To the best of our knowledge, this is the first study indicating that the process of aging induced worse insulin- and IGF-1-mediated vasorelaxation in SHRs, which was mainly attributed to the impaired activation of NOS and PI3K and reduced NO production. Moreover, the serum level of oxidative stress was significantly increased, while the antioxidant activities were significantly reduced in the older SHRs when compared with the younger SHRs.

Insulin and IGF-1 play crucial roles in regulating the patho-physiological function of the cardiovascular system [21]. Several studies have indicated that vascular actions of insulin and IGF-1 are impaired in some cardiovascular disorders, such as hypertension and diabetes [16,22]. Both insulin and IGF-1 mediate vasoactive responses through the endothelium-derived NO production mainly via the PI3K/NOS signaling pathway [22,23,24]. Our earlier study reported that the insulin- and IGF-1-induced vasorelaxation was impaired in aging hypertensive rats when compared with the age-matched normotensive rats [25]. In the present study, we further found that the 48wk-SHR group showed significantly worse vasorelaxation mediated by insulin and IGF-1 than the 24wk-SHR group (*p* < 0.05). However, no significant differences in the vasorelaxation mediated by insulin and IGF-1 existed between the 24wk-WKY and 48wk-WKY groups. Moreover, in the endothelium-denuded rings, these vasorelaxing responses were diminished, and no significant differences were found among the four groups. These findings suggested that the process of aging additively affected the endothelium-dependent vasorelaxation mediated by insulin and IGF-1 in the hypertensive groups but not in the normotensive groups. A previous study indicated that aging caused significant endothelial dysfunction, such as the decreased arterial vasorelaxing responses to insulin, in the Sprague–Dawley (SD) rats. In addition, aging increased the insulin resistance-related hypertensive response in SD rats. This implied that the endothelial dysfunction in response to insulin could play an important role in the development of aging-related hypertension [26]. Consistently, we found that the coexistence of aging and hypertension induced more severe endothelial dysfunction in response to insulin and IGF-1 in older SHRs. Furthermore, after the administration of L-NAME or wortmannin, the insulin- and IGF-1-mediated vasorelaxation was significantly reduced and not significantly different among the four groups. This indicated that the worse insulin- and IGF-1-mediated vasorelaxation in older SHRs was mainly attributed to the impaired activation of NOS and PI3K. These results supported the findings from previous studies that revealed that impaired arterial vasorelaxation to insulin and IGF-1 was related to the decreased eNOS expression and endothelial NO release in hypertension [16,17,18].

The processes of aging and hypertension negatively influence the endothelial function, respectively. Until now, various hypotheses have been proposed to explain the reduction of the endothelium-dependent vasorelaxation in the process of arterial aging: a decreased number of vasodilator receptors in the endothelium, a diminished capability to generate NO by the endothelium, and a reduction in guanylate cyclase activity in vascular smooth muscle cells [27]. In addition, impaired NO production and bioavailability are considered as the key features of endothelial dysfunction and may precede the increase in blood pressure in hypertension. This may be related to the imbalance of endothelium-derived vasoconstrictive and vasodilatory substances, with a shift toward greater vasoconstriction and thrombosis formation [28,29]. In this study, our results demonstrated that the 48wk-SHR (older) group had significantly decreased NO production compared with the 24wk-SHR (younger) group. It indicated that the greater decreases in NO production contributed to the worse impairments of endothelium-dependent vasorelaxation mediated by insulin and IGF-1 in the coexistence of aging and hypertension. These results were consistent with the previous studies that reported an impairment of NO bioavailability in aging blood vessels. They pointed out that impaired NO bioavailability was partly due to the aging-related reduction in BH_4_ availability or arginase upregulation by modulating L-arginine availability [30,31,32]. Several studies have found that impaired NO production has been found in aging and hypertension, respectively [33,34,35]. Moreover, in our laboratory, some results indicated that the eNOS expression was significantly reduced in aortic tissues of hypertensive rats, leading to the decreased NO production [34]. In agreement with a study in human subjects, they found that the endothelium-dependent vasodilation to acetylcholine was declined with increasing age in hypertensive patients, mediated mainly by the age-related reduction of NO availability [36].

As well-known in the last centuries, oxidative stress has been considered to promote endothelial dysfunction and lead to vascular damage, which is involved in the pathogenic mechanism of hypertension [9,37,38]. Moreover, with increasing age, the oxidative stress probably contributes to impaired NO bioavailability and endothelium-dependent vasorelaxation in the elderly [10]. The level of oxidative stress increases as a result of exceeding the production of ROS without enough compensation of antioxidant activity. Several sources of ROS production include the up-regulation of the oxidant enzyme nicotinamide adenine dinucleotide phosphate (NADPH) oxidase, uncoupling eNOS (due to lacking a cofactor of production, i.e., tetrahydrobiopterin; BH_4_), and increased mitochondrial synthesis during oxidative phosphorylation of the electron transport chain [38]. As a result, with aging and hypertension, excessive oxidative stress could be the key mechanism influencing impaired NO bioavailability and endothelium-dependent vasorelaxation. In aging, oxidant theory was proposed as the cumulative result of oxidative damage to the cells and tissues of the body that arises primarily as a result of aerobic metabolism with an imbalance between the production and degradation of ROS [12]. Oxidative stress may induce uncontrolled lipid peroxidation, which in turn gives rise to cell injuries via DNA damage, leading to endothelial dysfunction [39]. MDA is a well-established biomarker of oxidative stress to lipids. On the other hand, decreased TAC levels, reflecting increased oxidative stress, might be the reason for increased total lymphocyte DNA damage in hypertensive patients [40]. Several studies have reported the remarkable increase in MDA but decrease in TAC and catalase activity in aging, relating to risks of cardiovascular diseases and all-cause mortality [41,42,43]. Moreover, in hypertension, these oxidant markers are well detected in similar trends with aging, but there is a lack of information regarding the synergistic effect of aging on oxidative stress in the hypertensive population [9]. In the present study, our results indicated that the older hypertensive group showed the highest MDA concentration and the lowest antioxidant activities (i.e., catalase activity and TAC) among the four groups. These findings suggested that the process of aging additively affected the status of oxidative stress in hypertension.

The reason for the above findings might be that the continuous increase in ROS production, along with a concomitant disruption in redox balance, leads to a state of chronic inflammation [44]. Furthermore, hydrogen peroxide (H_2_O_2_) activates nuclear factor kappa-B (NF-kB), which augments the transcription of proinflammatory genes, leading to increased expression of tumor necrosis factor alpha (TNF-α), interleukin 6, chemokines, and adhesion molecules, implicated in the development of atherogenesis [45]. In addition to inflammation, the renin-angiotensin-aldosterone system (RAAS) has been reported to contribute to the age-associated increase in NO inactivation. With aging and hypertension, the RAAS activity and the concentration of angiotensin II could be upregulated, consequently increasing the production of ROS by activating NADPH oxidase [46]. Moreover, sirtuin 1 (SIRT1), a nicotinamide adenosine dinucleotide (NAD)-dependent deacetylase, may cause synergetic effects of aging on endothelial dysfunction in hypertension since the age-related loss in SIRT1 is associated with increased senescence. The activation of SIRT1 has been found to upregulate members of the class O of forkhead box (FOXO) transcription factors and eNOS to reduce oxidative stress and vessel reactivity alteration [47]. Furthermore, Poudel and co-workers pointed out that age-induced decreases in IGF-1 signals were associated with increased oxidative stress. The supplementation of IGF-1 in aged rats could protect from oxidative damage and restore levels of SOD, glutathione peroxidase, and catalase. Additionally, the IGF-1-mediated activation for the PI3K-AKT/FOXO pathway upregulated the transcription of antiapoptotic genes [48]. These signaling pathways play important roles in counteracting aging and hypertension. However, the potential mechanisms that additively influence the ROS and antioxidant activity for these impairments in aging hypertensive rats need to be further clarified.

## 5. Conclusions

**Figure 6:**
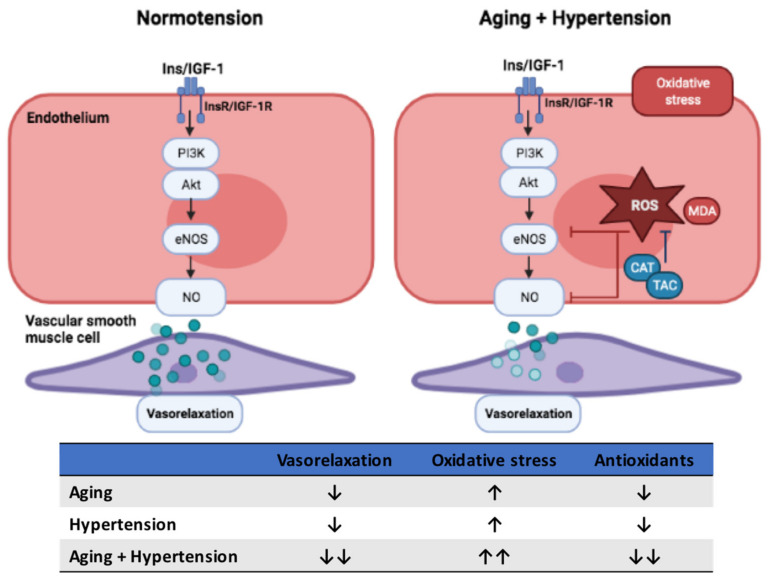
The proposed scheme demonstrating potential mechanisms involved in the impaired insulin- and IGF-1-mediated vascular relaxation in age-related hypertension. The excessive production of ROS and antioxidant deficiency in either aging or hypertension reduced NO production and consequently impaired insulin- and IGF-1-mediated vascular relaxation. Furthermore, our finding indicated that the process of aging additively affected insulin- and IGF-1-mediated endothelial dysfunction in aging-related hypertension, which was partly attributed to the reduced NO production and imbalance of oxidative and antioxidant activities in hypertension. Ins, insulin; IGF-1, insulin-like growth factor-1; R, receptors; PI3K, phosphoinositide 3-kinase; Akt, protein kinase B; eNOS, endothelial nitric oxide synthase; MDA, malondialdehyde; CAT, catalase activity; TAC, total antioxidant capacity; ↑, increasing; ↑↑, more increasing; ↓, decreasing; ↓↓, more decreasing.

This study demonstrated that the process of aging additively affected insulin- and IGF-1-mediated endothelial dysfunction mainly through impairing the PI3K-NOS-NO pathway in SHRs. Furthermore, this was partly attributed to the reduced NO production and imbalance of oxidative and antioxidant activities in hypertension. Integrating with previous studies, our findings provided a better understanding of the potential mechanisms involved in the impaired insulin- and IGF-1- mediated vascular relaxation in age-related hypertension (as shown in Figure 6). Future studies are recommended to confirm the roles of oxidative stress and antioxidants for insulin- and IGF-1-mediated cardiovascular dysfunction in the elderly population with hypertension.

## Figures and Tables

**Table 1:** General characteristics.

	24wk-WKY	48wk-WKY	24wk-SHR	48wk-SHR
Body weight (g)	378.13 ± 1.89	401.00 ± 2.90 *	369.63 ± 5.47 ^#^	393.63 ± 5.10 *^,¶^
Heart rate (bpm)	302.00 ± 3.86	301.75 ± 4.41	380.50 ± 4.46 *^,#^	393.69 ± 10.53 *^,#^
SBP (mmHg)	121.56 ± 0.85	121.25 ± 1.09	194.81 ± 2.42 *^,#^	195.88 ± 0.99 *^,#^
DBP (mmHg)	96.63 ± 0.58	95.56 ± 1.10	151.25 ± 1.38 *^,#^	154.25 ± 1.50 *^,#^
MAP (mmHg)	109.75 ± 1.15	108.56 ± 0.93	168.63 ± 1.04 *^,#^	168.44 ± 1.12 *^,#^
Glucose (mg/dL)	91.75 ± 2.05	99.00 ± 3.16	123.00 ± 3.51 *^,#^	119.63 ± 5.84 *^,#^
Insulin (μg/L)	0.33 ± 0.05	0.35 ± 0.07	0.43 ± 0.05	0.65 ± 0.08 *^,#,¶^
HOMA-IR	1.86 ± 0.29	2.06 ± 0.39	3.21 ± 0.34 *^,#^	4.73 ± 0.51 *^,#,¶^

Values are means ± SEM. Abbreviations: SBP, systolic blood pressure; DBP, diastolic blood pressure; MAP, mean arterial pressure; HOMA-IR, the homeostatic model assessment of insulin resistance; 24wk, 24-week-old; 48wk, 48-week-old; WKY, Wistar–Kyoto rat; SHR, spontaneously hypertensive rat. * *p* < 0.05, significant differences from 24wk-WKY; ^# ^*p* < 0.05, significant differences from 48wk-WKY; ^¶ ^*p* < 0.05, significant differences from 24wk-SHR; *n* = 8 in each group.

## Data Availability

The raw data supporting this study will be made available by the corresponding author upon reasonable request.
